# Current understanding of the role of microRNAs in spinocerebellar ataxias

**DOI:** 10.1186/2053-8871-1-7

**Published:** 2014-08-07

**Authors:** Edyta Koscianska, Wlodzimierz J Krzyzosiak

**Affiliations:** Department of Molecular Biomedicine, Institute of Bioorganic Chemistry, Polish Academy of Sciences, Noskowskiego 12/14 Str, 61-704 Poznan, Poland

**Keywords:** Ataxin, PolyQ expansion, TREDs, miRNA, Target validation, Luciferase assay

## Abstract

The number of studies highlighting the role of microRNAs (miRNAs) in human physiology and diseases is growing, but many miRNA-driven regulatory mechanisms remain elusive. A proper understanding of the exact functions of individual miRNAs and their interaction with specific targets is vitally important because such knowledge might help cure diseases for which no effective treatment currently exists. Herein, we present current views on the role of the miRNA-mediated regulation of gene expression in the case of select spinocerebellar ataxias (SCAs) and their potential involvement in the pathogenesis of these diseases. Specifically, we summarize published data showing the known links between miRNAs and CAG repeat-dependent SCAs. Moreover, using the example of SCA type 3 (SCA3), we refer to the issue of prediction and validation of miRNA targets, and we demonstrate that miR-181a-1 may regulate the 3′-UTR of the *ATXN3* gene.

## Introduction

Several spinocerebellar ataxias (SCAs) belong to trinucleotide repeat expansion disorders (TREDs), which are a relatively large group of incurable dominantly inherited neurological diseases. More specifically, six SCAs belong to polyglutamine (PolyQ) diseases and are triggered by an expansion of the glutamine-coding CAG repeat, which is responsible for polyQ tract formation. A long polyQ stretch can elicit numerous aberrant cellular processes that lead to the degeneration and death of neuronal cells.

MicroRNAs (miRNAs) are potent regulators of gene expression. They act at the post-transcriptional level by inhibiting protein synthesis that occurs with or without transcript degradation. It appears that these small RNAs are engaged in a wide range of physiological and pathological activities in cells; therefore, their function and contribution to the pathogenesis of SCAs is of special consideration.

## Review

This review summarizes the current knowledge regarding the putative involvement of miRNAs in the pathogenesis of CAG repeat-dependent SCAs and the experimentally proven associations between specific miRNAs and these diseases that have been reported. Moreover, it addresses some aspects of miRNA target prediction and validation using the example of our own research on the regulation of the ataxin-3 transcript.

### Brief characteristics of CAG repeat-dependent SCAs and miRNA-mediated regulation of gene expression

There are six SCAs (SCA-1, 2, 3, 6, 7, and 17) among the polyQ diseases [1–3], and their common denominator is the expression of CAG repeats of abnormal length in the open reading frame (ORF) of the ataxin (*ATXN*) genes. The pathogenic glutamine repeat threshold in most of these SCAs is approximately 35, except for SCA3 and SCA6, in which cases the pathogenic repeat range starts from 50 and 21, respectively. The ataxias that fall under the category of polyglutamine diseases have similar clinical presentations; however, molecular studies are necessary to distinguish particular SCA types. Current views on the identified molecular processes causing or modulating the neurodegenerative phenotype in spinocerebellar ataxias have been discussed recently and reviewed thoroughly [4].

The normal function of the polyglutamine proteins implicated in SCAs seems to be quite disparate and may be succinctly defined as follows: ataxin-1 and ataxin-7 play a role in transcription regulation [5–7], ataxin-2 is involved in RNA metabolism and endocytosis processes [8, 9], ataxin-3 interacts with transcriptional components and is responsible for de-ubiquitination and transcription regulation [10], ataxin-6 is linked to Ca^+^ signaling/homeostasis [11], and ataxin-17 is linked to general transcription (TFIID complex) [12] (reviewed in [4]).

The expanded CAGs may trigger pathogenic effects based on three types of mechanisms: (1) toxic RNA gain-of-function, (2) toxic protein gain-of-function, or (3) both transcript and protein loss-of-function [3, 13, 14]. The toxic protein gain-of-function mechanism explains neurodegeneration as the result of the expression of glutamine-rich proteins that can misfold and form aggregates, typically in nuclear inclusions, and eventually result in neuronal dysfunction and loss [15–17]. More recently, however, mutant CAG repeat transcripts have also been shown to contribute to the pathogenesis of polyQ diseases. Specifically, aberrant alternative splicing, transcript nuclear transport and export, RNA interference, and nucleolar stress resulting in apoptosis have been reported to be involved in CAG repeat RNA toxicity (reviewed in [18, 19]).

To date, more than 2,500 mature human miRNAs have been deposited in the miRNA registry (miRBase, Release 20) [20]. These important molecules are endogenous short RNAs that downregulate gene expression by imperfect pairing with complementary sites within transcript sequences; miRNAs suppress their targets’ translation, stimulate their deadenylation and degradation, or induce target cleavage (reviewed in [21]). Regarding the canonical biogenesis process, human miRNAs are generated by two RNase III endonucleases (Drosha and Dicer) that act sequentially in the nucleus and the cytoplasm (reviewed in [22–25]). The ribonuclease Drosha, acting together with the DGCR8 protein within the complex named Microprocessor, cleaves primary miRNA transcripts (pri-miRNA) into pre-miRNA precursors that are approximately 60 nucleotides long, while the ribonuclease Dicer processes pre-miRNAs into mature miRNA duplexes that are approximately 22 nucleotides long. Both canonical and non-canonical miRNA biogenesis pathways, the assembly of the miRNA-induced silencing complex (miRISC), and various intricate miRNA-mediated mechanisms of gene expression regulation have been recently discussed in detail [26].

Generally, the level of the polyQ-expanded protein is among the factors that contribute to disease severity [27–30]; however, the proposed cellular mechanisms that regulate protein levels remain to be fully characterized. miRNAs that are the key regulators of protein dosage in cells must therefore be directly linked with the pathogenesis of SCAs. The roles of miRNAs in the development and regulation of the nervous system and in a wide array of disorders of the nervous system have been proven in many studies (reviewed in [31–41]). Several miRNAs have been shown to target different neurodegenerative disease-related proteins and modulate their concentration in cells (e.g., BACE1, ataxin-1 or α-synuclein in the case of Alzheimer’s disease (AD), SCA1 and Parkinson’s disease (PD), respectively ([42, 43] and references therein)). Moreover, it was demonstrated that the dysfunction of the brain-enriched miRNAs that specifically target and regulate the expression of disease-associated genes may lead to neurodegeneration and that disease-related proteins interact with the miRNA machinery [44–46]. Therefore, it is of growing importance to address the plausible involvement of miRNAs in the pathogenesis of particular SCA types and to identify the activities of individual miRNAs on SCA disease-coding genes.

### Links between miRNAs and CAG repeat-dependent SCAs

Specific links between miRNA regulation and CAG repeat-dependent SCAs have been described in several studies (reviewed in [47]) (Table 1). However, most of hitherto published reports focus on the role of miRNAs in the most common SCAs, namely SCA1 and SCA3.

**Table 1:** **Reported changes in miRNA expression in SCAs and their links with polyQ toxicity**

Observed changes	miRNA prediction	Experimental methods	Experimental models	Refs.
**SCA1**				
miR-19, −101 and −130 downregulate the *ATXN1* gene	Candidate miRNAs were identified using the PicTar algorithm.	Transfection with miRNA duplexes and their specific 2′-O-methyl inhibitors followed by western blot and RT-PCR analyses. miRNAs transfected to cells either individually or collectively.	MCF7 (highly express endogenous ATXN1), HEK293T, HeLa and NIH3T3 cell lines	[48]
	Eight miRNAs were chosen for further examination based on the number of target sites in the ATXN1 transcript and their neuronal expression.			
		Luciferase reporter assays (Promega) with vectors carrying fragments or full length human ATXN1 3′-UTRs.	HeLa cell line	
		miRNA levels and their expression patterns in mouse cerebella were assessed by northern blot analysis and *in situ* hybridization with LNA probes (Exiqon).	C57/B6 WT mouse	
		Cell death assays with mutant ATXN1deprived of target sites.	HEK293T cell line	
miRNA expression upregulated in SCA1 patients; the increase more prominent in the cortex samples	Regulatory RNA network and TargetScan prediction algorithms.	miRCURY LNA human microRNA Array (Exiqon).	Human cerebellum and cortex, SCA1 patients and healthy controls	[50]
miR-144 slightly downregulated in SCA1 cerebellum but strongly induced in the cortex		qRT-PCR, TaqMan miRNA assays (Applied Biosystems) for miR-144 and miR-101.		
Upregulated miRNAs predicted to target ATXN1, e.g., miR-101, -130a, -19a, -302		ATXN1 mRNA and protein levels analyzed with RT-PCR and western blot.		
miR-144 and −101 downregulate ATXN1 expression, miR-25 does not affect ATXN1 levels		Overexpression of miRNA duplexes and miRNA inhibitors followed by western blotting.	HEK293T cell line	
		Luciferase assays (Promega) with full-length ATXN1-3′-UTR-hLuc reporters and miRNA duplexes or 2′-O-methyl modified masking oligos. miRs 144 and 101 tested separately and in combination.		
No evidence for a statistically significant difference in miRNA expression. A trend to overexpression of miR-33-5p, −34-5p and -92a-5p	-	Illumina Hi-Seq 2000, small RNAs from fly heads	*Drosophila* models: UAS-Atxn1-82Q and UAS-Atxn1-30Q	[56]
34 miRNAs upregulated; 14 at both time points, 15 at the 4-week time point only and 5 at the 12-week time point		miRCURY LNA all species microRNA arrays (Exiqon)	Cerebellar RNA from SCA1 BO5 transgenic mice [82Q] analyzed at two time points (at 4 and 12 weeks of age)	[53]
		Individual miRNAs analyzed by qRT-PCR with TaqMan assays (miRs 150, 335, 23a, 24 and 143)		
12 miRNAs downregulated; 1 miRNA at both time points, 4 and 7, respectively at the 4- and 12-week time points only				
miR-150 levels increased in cerebellar Purkinje neurons and slightly decreased in granule cells		*In situ* hybridization using dig-labeled DNA-LNA probes (Exiqon)	SCA1 BO5 transgenic mice - Purkinje neurons, granule cells	
A concomitant increase in miR-150 and decrease in *Rgs8* and *Vegfa* levels	TargetScan; *Rgs8* and *Vegfa* identified as targets of miR-150	Quantitative PCR and immunohistochemical analyses	SCA1 cerebella, Purkinje neurons	
A dose-dependent decrease in *Vegfa* expression induced by the increased miR-150 activity		Transient transfection with *MirVana* miR-150 miRNA mimics followed by qPCR and WB. Luciferase assays (Promega) with wild-type Vegfa-3′UTR and miR-150 mimic.	Mouse Neuro2A	
**SCA2**				
Upregulation of *bantam* and miR-12	-	Confocal microscopy	*Drosophila*, transgenic recombinant flies, wing imaginal discs cells	[60]
**SCA3**				
*bantam* miRNA is a downstream modulator/suppressor of polyQ toxicity	-	Phenotype mutants comparison analysis, TUNEL assays, immunostaining and western blots	*Drosophila dcr-1* mutants, fly eyes, HeLa cells with normal and pathogenic Ataxin-3 treated with siRNA targeting *dicer* mRNA	[57]
No evidence for a statistically significant difference in miRNA expression. A trend to a decrease in miR-1-3p and an increase in miR-100-5p, −33-5p and 92a-5p levels.	-	Illumina Hi-Seq 2000, small RNAs from fly heads	*Drosophila* models: UAS-Atxn3-70Q and UAS-Atxn3-19Q	[56]
miR-34b is upregulated, and miR-25, −125b, −29a are downregulated in SCA3 patients. Expression of miR-25 and -125b was associated with the course of disease.	miRbase, TargetScan and micro.org were used to search for miRNA binding sites in the human ATXN3 3′-UTR	miRCURY LNA human miRNA Array (Exiqon) (v.14.0), validation miRNA expression by qRT-PCR (Applied Biosystems)	Blood samples obtained from SCA3 patients (35) and control individuals (25)	[61]
**SCA7**				
No evidence for a statistically significant difference in miRNA expression. A trend to an increase in miR-33-5p and -92a-5p levels and a decrease in miR-375-3p	-	Illumina Hi-Seq 2000, small RNAs from fly heads	*Drosophila* models: UAS-Atxn7-102Q and UAS-Atxn7-10Q	[56]

### miRNAs in SCA1

A study on SCA1 was the first to reveal that some miRNAs can regulate the expression of target transcript mRNA containing a CAG repeat expansion. More specifically, that study showed that miR-19a, miR-101, and miR-130a co-regulate the 3′-UTR of ATXN1 through the inhibition of ATXN1 translation [48]. The gene coding for human *ATXN1* has a long 3′-UTR region (approximately 7 kb), which implies an important role in the post-transcriptional regulation governed by miRNAs. Moreover, cerebellar degeneration and ataxia in mice was reported to be interconnected with the impairment of miRNA biogenesis in Purkinje cells [49]. In a reporter assay, which is the simplest and most straightforward method of validation of miRNA-mRNA interactions, miRs 19a, 101, and 130a were shown to downregulate the expression of ATXN1 [48]. Interestingly, miR-101 affected both the mRNA and protein levels, whereas miR-19a and miR-130a decreased the protein levels only. Although these miRNAs exerted their effect in different cell lines when transfected individually, a markedly stronger effect was observed when miRs 19a, 101 and 130a were transfected together and cooperated in the regulation of their target. Moreover, inhibiting the endogenous expression of all three miRNAs in HEK293T cells with specific inhibitors and the mutagenesis of miRNA target sites in the 3′-UTR of a hATXN1[86Q] expression vector enhanced the cytotoxicity of the mutant ATXN1 protein and significantly reduced cell viability. Taken together*,* these findings show that miRNA-mediated posttranscriptional regulation of the *ATXN1* gene may modulate SCA1-related neuropathology by affecting protein levels.

Another key example of the potential involvement of miRNAs, especially miR-144, in brain aging and SCA1 pathogenesis has been provided by a genome-wide microarray analysis [50]. This study revealed a global age-related miRNA deregulation in the cortex and cerebellum of humans, chimpanzees and rhesus macaques. Interestingly, the vast majority of miRNAs in the examined samples were downregulated, and only a small fraction of miRNAs were found to be upregulated. A highly conserved miR-144 was found to be related to the aging process but regulated differently between species. Its expression was decreased in the human cortex but was consistently induced in the aging cerebellum and cortex of nonhuman primates. Moreover, a subsequent analysis of human samples showed a widespread activation of miRNA expression in the cortex and cerebellum of SCA1 patients relative to the miRNA expression in the brains of healthy individuals. The level of miR-144 that was predicted to bind to the ATXN1 3′-UTR was elevated in the cortex. Other miRNAs (e.g., miR-101, −130a, −19a, −302) predicted to target ATXN1 for degradation were also found to be upregulated. Next, the potential regulation of the ATXN1 transcript by the candidate miRNAs was examined using miRNA overexpression and inhibition as well as reporter assays. Of the three miRNAs tested, miRs 144 and 101 considerably decreased the endogenous ATXN1 protein levels (miR-25 activity was not observed). Similarly, these two miRNAs reduced reporter gene expression when tested either separately or collectively. Overall, it was concluded that the activation of specific miRNAs could serve to reduce the cytotoxic effect of the expanded mutATXN1 and that miRNA deregulation may be a risk factor for disease development. Protective functions for miRNAs have been also proposed by others [33, 37, 51, 52].

Global profiling of miRNA expression in a mouse model of SCA1 was performed to study alterations in miRNA expression in the cerebellum at both pre-symptomatic and symptomatic stages of pathogenesis [53]. More specifically, the analysis revealed changes in the levels of 46 miRNAs in BO5 SCA1 transgenic mice [82Q], and a number of miRNAs displayed altered expression patterns before the onset of clear phenotypes. Mice were analyzed at two time points, i.e., 4 and 12 weeks. Of the miRNAs that were changed in the SCA1 mouse cerebella, 34 displayed increased expression; 14 of these miRNAs exhibited increased expression for both time points analyzed, and 15 and 5 exhibited increased expression at only the 4- or 12-week time points, respectively. In contrast, the miRNA expression level was decreased for 12 miRNAs, one at both time points and 4 and 7 at the 4- and 12-week time points, respectively. Importantly, the observed changes in miRNA expression (e.g., miR-150 and miR-335) were found immediately after the start of mutant Atxn1 expression in SCA1 mice and prior to phenotypic onset, showing that the deregulation of miRNA expression in the cerebellum of transgenic mice contributes to the early pathogenesis of SCA1. Moreover, in line with the fact that the expression of the mutAtxn1 protein is restricted to cerebellar Purkinje cells in SCA1 transgenic mice, elevated miR-150 expression was observed selectively in Purkinje neurons. In addition, the levels of two transcripts that are predicted targets of miR-150, *Rgs8* and *Vegfa*, were reduced. The latter target was further validated in Neuro2A cells using miR-150 mimics and reporter assays; analyses of both types confirmed the functionality of binding sites for miR-150 that are present in *Vegfa* mRNA. Collectively, these data demonstrated that the expression levels of mutAtxn1 and miR-150 together with its target mRNA are highly interconnected. It was proposed that the mutAtxn1 de-represses activity at miRNA promoters, leading to an increase in miRNA levels, or acts through yet unknown a gain-of-toxic function mechanism. Therefore, evidence that miRNA misregulation during the critical post-natal period may underlay SCA1 pathogenicity was provided. Moreover, in this work, a promising therapeutic approach based on the RNAi-mediated suppression of mutant Atxn1 was presented. This therapeutic intervention for SCA1 has been developed further by the same group and described elsewhere [54].

The miRNA deregulation at the beginning of the pathological process has been also investigated in *Drosophila* transgenic model of SCA1 with the use of deep sequencing [55]. Specifically, two RNA samples from flies and one control were analyzed. The expression of miRNAs was evaluated at an early stage of the pathological process, 3 days after the induction of toxic proteins. Many of the selected miRNAs displayed low expression or highly variable expression between the tested samples. Although a trend toward the overexpression of miR-33, miR-34 and miR-92a was observed, no evidence for a statistically significant difference in miRNA expression at this early stage of the disease was found. Therefore, these results suggest that the toxicity in SCA1 in the early stages of the pathological process and the deregulation of miRNA expression most likely are not related.

### miRNAs in SCA3

The miRNA *bantam* (*ban*) was identified as a downstream modulator/suppressor of polyQ toxicity in a *Drosophila* model of SCA3 that prevents degeneration [56]. Upregulation of the *ban* miRNA suppressed degeneration and diminished the toxicity of ataxin-3, which is the disease-causing protein for SCA3. Suppressing *ban* activity was connected with neither a change in the level of the pathogenic protein nor its accumulation and was shown to occur downstream of the polyQ protein accumulation. Moreover, reduced miRNA processing due to the knockout of Dicer1 enhanced the neurodegeneration induced by ataxin-3 in both flies and human cells by modulating pathways that normally contribute to polyQ toxicity [56]. The protective activity due to miRNAs was found to be specific because siRNA-dependent pathways did not modulate polyQ toxicity. Therefore, this research adds another piece of corroborating evidence that miRNA pathways dramatically modulate polyQ-induced neurodegeneration.

A protective role of miRNAs in age-associated processes and polyQ disorders was further supported by demonstrating that miR-34b mitigates the toxicity of ataxin-3 in *Drosophila* [57]. Specifically, the upregulation of miR-34 in this fly model extended the median lifespan and reduced the neurodegeneration induced by human polyQ disease protein.

*Drosophila* transgenic model of SCA3 has been also used to investigate deregulation of miRNA expression by deep sequencing, as described for SCA1 with regard to the same analysis [55]. The study conducted at the beginning of the pathological process revealed a trend toward a decrease in miR-1 and an increase in miR-33, miR-92a and miR-100 levels, however, the observed changes in miRNA expression were not statistically significant.

Most recently, it has been demonstrated that miRNAs are useful biomarkers for SCA3, as specific changes in regulatory miRNA levels were identified in the serum obtained from patients suffering from this disease [58]. The microarray analysis validated by qRT-PCR revealed that miR-25, miR-125b, miR-29a and miR-34b were differentially expressed in SCA3 patients. Additionally, miR-29a and miR-34b showed the most dramatic changes in their expression levels, whereas the expression of miR-25 and mir-125b was associated with the course of disease. The latter miRNAs, miRs 25 and 125b, were found to bind to the 3′-UTR of the *ATXN3* gene. All miRNAs proposed by Shi *et al.* as potential biomarkers were earlier reported to be connected with neuronal physiology and pathology in terms of neurodegeneration [59–63]. Moreover, mir-34b was previously shown to be elevated in the plasma of pre-manifest Huntington’s disease (HD) patients and predicted to be a protective factor for HD [60]. These findings, which advocate the use of serum miRNAs as biomarkers, are of potential value in the diagnosis of patients with neurodegenerative disorders.

### miRNAs in other CAG repeat-dependent SCAs

The involvement of miRNAs in the pathogenesis of SCA7 has been investigated in *Drosophila* transgenic model of this disease, along with studying their role in SCA1 and SCA3 [55]. As aforementioned, global profiling of miRNA expression did not show significant changes in miRNA levels at an early stage of the pathological process in the examined SCAs. In the case of SCA7 the level of miR-33 and miR-92 tended to be higher and the level of miR-375 was found to be lower but these differences were not statistically significant. Therefore, no important associations between the toxicity induced by the expansion of CAG repeats in SCA7 and the deregulation of miRNA expression have been reported.

With regard to other SCA types, the link between neurodegeneration and miRNAs was demonstrated by showing that the gene encoding TATA-binding protein (TBP), which is mutated in SCA type 17 (SCA17), is downregulated by miR-146a [64]. Finally, some pathogenic proteins, such as ataxin-2, might be required for miRNA functioning; it was shown that the ataxin-2 protein, which interacts with Ago1, impairs the repressive activity of several miRNAs [65].

### Summary of miRNA-mediated gene regulation in SCAs

**Figure 1:**
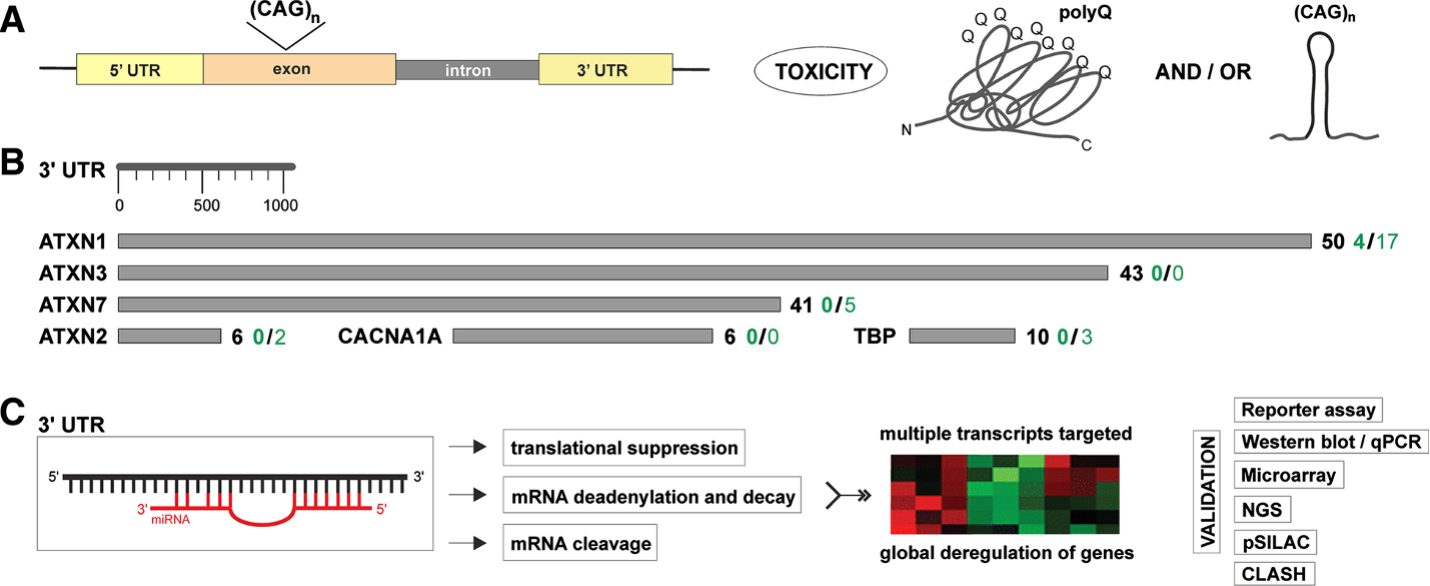
**Potential contribution of miRNAs to the pathogenesis of CAG repeat-dependent SCAs. A)** The schematic presentation of the CAG expansion in the *ATXN* genes and CAG expansion-induced toxicity factors. **B)** The graphical presentation of the 3′-UTRs of SCA disease-causing genes. Grey rectangles represent appropriate 3′-UTRs. Their length is delineated and a scale of 1,000 nt is shown. Black numbers indicate the number of sites for miRNA families broadly conserved among vertebrates predicted by the TargetScanHuman algorithm (Release 6.2) [70]. Green numbers show the number of validated miRNA-target interactions collected in the miRTarBase (Release 4.5) [85]; bold green numbers and unbolded ones denote the number of interactions validated by methods providing strong and less strong evidence, respectively. **C)** Mechanisms of miRNA-mediated control of gene expression. The main regulatory activities of miRNAs and global deregulation of multiple genes are schematically presented. Additionally, essential methods used for validation of miRNA-mRNA interactions are listed.

All of the above-mentioned findings suggest that miRNA pathways can modulate polyQ-induced neurodegeneration but that the pathogenesis of polyQ diseases can also influence miRNA expression. It has been proven that miRNAs are implicated in the pathogenicity associated with SCAs at many levels of different pathological processes; however, miRNA specific functions remain largely unknown. Moreover, the question persists, how exactly miRNAs affect CAG repeat-mediated neurodegeneration and whether such miRNA activity is similar in all SCA types. A concept relating miRNAs and ataxias could be built on the known miRNA mode of action (Figure 1). The miRNA-mediated regulation of gene expression may be exerted by different mechanisms (reviewed in [26]), eventually leading to the reduction in levels of both factors responsible for the pathogenesis of polyQ diseases, i.e., mutant proteins and toxic transcripts [66]. The 3′-UTR regions of CAG repeat-dependent SCAs vary drastically in length, which may be important with regard to the fact that miRNA binding sites are mostly located in 3′-UTRs of target genes. Although 3′-UTR lengths did not always correlate with the number of miRNA binding sites, the ATXN1 3′-UTR being the best-studied in terms of miRNA-mediated regulation is the longest and contains the greatest number of predicted miRNA binding sites [67]. Apart from direct regulation of genes triggering particular SCAs, miRNA may be also engaged in the pathogenesis of these diseases indirectly by targeting multiple transcripts and affecting expression of numerous proteins. All in all, miRNAs regulatory potential contributes to the pathogenesis of CAG repeat-dependent SCAs.

### Prediction and validation of miRNA targets in SCA3

Identifying biologically relevant miRNA targets is of paramount importance and involves the reliable prediction of miRNA-mRNA interactions and the positive validation of functionality of these interactions. Many different algorithms have been developed to predict miRNA-mRNA binding; computational tools such as miRANDA [68], PicTar [69], TargetScan [70], RNAhybrid [71] or PITA [72] have been successfully used for more than a decade, while the more recently invented programs miRco [73] and MREdictor [74] are currently coming into use. Similarly, various approaches for the experimental verification of predicted miRNA-mRNA interactions have been reported to date (reviewed in [75–77]). The most straightforward method for the verification of miRNA function is a transfection of cells with miRNA mimics or miRNA inhibitors, followed by quantitative analyses of target mRNA and protein levels [78, 79]. There are also various high-throughput methods, such as the increasingly popular small RNA deep sequencing and microarrays as well as proteome and transcriptome analyses, which allow the identification of thousands of miRNA-target pairs [80, 81]. Moreover, the most advanced and modern methods are based on UV crosslinking and immunoprecipitation followed by deep sequencing and bioinformatic mapping of the reads (reviewed in [82]). The simplest and most reliable method, however, is the use of luciferase reporter assays because miRNA activity on reporter genes can be easily measured [69, 83]. Reporter systems enable the study of single specific miRNA-mRNA interactions (reviewed in [84]) and have proven to be convenient and reliable in many studies (data and references collected in [85]). Therefore, they serve as an efficient and routinely used strategy for the verification of individual miRNA-mRNA interactions. More than 4,000 miRNA-target interactions have been validated by reporter assays, the collection of which is considered to provide strong experimental evidence accumulated in the miRTarBase [85, 86], which is the database containing the largest amount of such validated interactions (51,460 in total).

**Figure 2:**
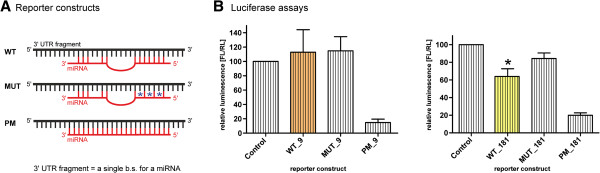
**Validation of the interaction between miRNAs and mRNAs. A)** Schematic representation of the standard reporter constructs used for the experimental validation of miRNA-mRNA interactions. WT, MUT and PM denote appropriate constructs; a construct bearing a wild-type potential binding site for the studied miRNA (WT), a construct with mutated binding site (MUT), and a construct with full complementarity (PM). **B)**. Regulation of the ataxin-3 transcript by miR-9-2 and miR-181a-1. Relative repression of luciferase expression for both miRNAs is shown. The standard errors were calculated from independent experiments. The asterisk indicates statistical significance (*p* value < 0.05).

SCA3 (also known as Machado-Joseph disease) is among the most common dominantly inherited ataxias [87–89]. The post-transcriptional miRNA-mediated regulation of the disease-causing ATXN3 expression has been described only in few reports [55, 56, 58] (Table 1). In our previous study, we performed an in-depth computational analysis of the miRNA interactions with all mRNAs derived from genes triggering hereditary neurological disorders known as trinucleotide repeat expansion diseases (TREDs) and showed that the gene mutated in the case of SCA3 may be subject to miRNA regulation [67]. From predicted potential interactions, we selected two for further experimental verification using a reporter system. Specifically, we have preliminarily validated a putative regulation of the ataxin-3 transcript by two miRNAs, miR-9-2 and miR-181a-1, by employing a luciferase assay and a set of reporter constructs as described previously [90, 91]. Briefly, synthetic oligonucleotides corresponding to sequences of single binding sites (b.s.) for the appropriate miRNAs (ATXN3 b.s. for miR-9-2 and miR-181a-1) were cloned into the pmirGLO Dual-Luciferase miRNA Target Expression Vector (Promega). This vector, which was specially designed for miRNA-mRNA interaction studies, is based on Promega dual-luciferase technology, with firefly luciferase (*luc2*) as the primary reporter for monitoring mRNA regulation and *Renilla* luciferase (*hRluc-neo*) as a control reporter for normalization. Typically, in a reporter system, three types of constructs are prepared; a wild-type (WT) construct bearing an intact binding site(s) for the studied miRNA, a construct with mutations in its binding site(s) (MUT), and a perfect match (PM) construct (Figure 2A). Mutations in the sequence corresponding to the miRNA seed disrupt the native pairing within the binding region of the candidate miRNAs, which provides a negative control, whereas perfect complementarity with miRNA sequences induces target degradation and provides a positive control.

To validate the interactions between our candidate miRNAs, miR-9-2 and miR-181a-1, and the ATXN3 transcript, we transfected HEK293T cells with either reporter carrying potential miRNA binding sites. Four constructs were transfected into cells and tested in parallel. These constructs were co-transfected with appropriate miRNA-coding plasmid vectors (System Biosciences) because our experimental system required miRNA overexpression. The transient transfection of cells with reporter constructs was followed by measuring the reporter activity, which enabled the validation of predicted miRNA-mRNA interactions. Specifically, we obtained considerable repression of the luciferase expression after the transfection of reporter constructs for miR-181a-1, whereas no repression was measured after the transfection of reporters for miR-9-2 (Figure 2B).

The reduction in luciferase activity in the case of the WT construct carrying b.s. for miR-181a-1 was reproducible and statistically significant (suppression to 64%). The PM construct repressed luciferase to a very low level, while the luciferase activity for the MUT construct showed efficient de-repression. In contrast, we did not observe any decrease in luciferase activity when the WT construct carrying b.s. for miR-9-2 was transfected to cells. As for the experimental procedures, enzymatic activities of firefly and *Renilla* luciferases were measured using a luminometer (Berthold Technologies) with the substrates and procedures from the Dual Luciferase Assay Kit (Promega). The values for firefly luciferase activity for every reporter construct were normalized to the corresponding values of *Renilla* luciferase activity to account for varying transfection efficiency. Relative expression values for all constructs were obtained by comparing their normalized luciferase activities with those of the control plasmid. Transfection was repeated 6 and 8 times for miR-9-2 and miR-181a-1, respectively.

Overall, using the luciferase reporter system, we have validated the interaction between miR-181a-1 and the 3′-UTR of ATXN3 mRNA positively and the interaction between miR-9-2 and the 3′-UTR of ATXN3 mRNA negatively. This is only a preliminary validation of miRNA-mRNA interactions with respect to SCA3 and needs to be elaborated farther, and it is an example of the utility of the reporter system in the search for miRNA targets. Nevertheless, this is the first evidence for the direct binding of miR-181a-1 to the 3′-UTR of the human *ATXN3* gene.

## Conclusions

Many studies have shown that miRNAs play a crucial role in the development and functional regulation of the nervous system, and the deregulation of miRNAs was postulated and proved in numerous neurological disorders of different etiology, including TREDs and even strictly polyQ diseases (reviewed in [47]). Modest but significant changes in miRNA expression were shown to influence severity and/or disease progression. Hence, the deregulation of miRNA expression is now being considered a hallmark of many diseases that has both prognostic and diagnostic value. All studies performed on the CAG repeat-dependent SCAs demonstrate changes in miRNA expression patterns in these disorders and, most importantly, suggest that miRNA pathways and polyQ-induced neurodegeneration are interdependent processes. There is accumulating evidence that miRNAs are important contributors to the pathogenicity associated with SCAs. Therefore, close attention should be paid to the therapeutic potential of miRNAs due to their regulatory activities. Both the miRNA-based therapy (e.g., the use of miRNA mimics, artificial miRNAs, miRNA inhibitors and sponges) as well as the implementation of miRNA-based biomarkers would be beneficial in the treatment of all SCAs. Moreover, in terms of therapy for these diseases particularly important is the fact that miRNAs may repress the levels of both toxicity factors, namely polyQ protein and mutant CAG repeat transcript. Of all the CAG repeat-dependent SCA types studied to date, however, most findings are related to SCA1 and, secondarily, to SCA3. The role of miRNAs in the pathogenesis of other CAG repeat-dependent SCAs and miRNA-specific functions remain largely unknown and require further investigation.
